# Chemical Profiling and Tyrosinase Inhibition Mechanism of Phenylethanoid Glycosides from *Corallodiscus flabellatus*

**DOI:** 10.3390/molecules30112296

**Published:** 2025-05-23

**Authors:** Hong-bo Deng, Yao Yao, Hai-zhou Li

**Affiliations:** Faculty of Life Science and Technology, Kunming University of Science and Technology, 727 Jingming South Road, Chenggong District, Kunming 650500, China

**Keywords:** *Corallodiscus flabellatus*, phenylethanoid glycosides, tyrosinase inhibition, UV adaptation

## Abstract

Alpine plants face intense ultraviolet (UV) radiation in high-altitude ecosystems, necessitating adaptive mechanisms like tyrosinase-mediated phenolic metabolism for UV protection. This study aimed to characterize the phenolic profile of *Corallodiscus flabellatus* (or *C. flabellata*) and elucidate its mechanistic interactions with tyrosinase under high-altitude environments with intense ultraviolet (UV) radiation. Two novel phenylethanoid glycosides (PhGs) and seven known compounds were isolated using silica gel, ODS, and preparative HPLC, with structures determined via NMR, HR-ESI-MS, and acid hydrolysis. Tyrosinase (EC 1.14.18.1) inhibition assays revealed divergent effects: compound **7** (containing a caffeoyl moiety) exhibited potent inhibition (IC_50_ = 0.23 μM), comparable to arbutin, while other PhGs displayed activation or biphasic responses. Molecular docking analysis demonstrated that compound **7** stabilized tyrosinase via π-π stacking with Phe264 and Cu^2+^ coordination, whereas activating compounds likely acted as substrates. These findings elucidate the dual regulatory function of PhGs, which activate tyrosinase to counteract acute ultraviolet-induced stress and inhibit its activity to attenuate oxidative overload, thereby advancing our understanding of alpine plant adaptation mechanisms.

## 1. Introduction

The reduced atmospheric density in plateau ecosystems results in intensified ultraviolet (UV) radiation compared to lowland regions. This increased UV exposure, due to the unique geographical and climatic conditions, presents challenges to the growth and development of plants. Alpine vegetation has evolved sophisticated adaptation mechanisms against UV insult, involving multi-tiered strategies: tyrosinase-mediated enzymatic regulation, enhanced biosynthesis of UV-absorbing secondary metabolites, morpho-anatomical modifications, and reinforcement of antioxidant defense systems. These survival-critical adaptations not only ensure species perpetuation but also maintain ecological equilibrium in fragile high-altitude biomes.

UV-B radiation (280–315 nm) exerts dualistic effects on plant physiology. While moderate exposure induces photomorphogenic responses that optimize light acclimatization, supra-threshold irradiation triggers genotoxic cascades through DNA lesion formation, photosynthetic apparatus impairment, and reactive oxygen species (ROS) overproduction [1]. To mitigate UV-B-induced oxidative stress, plants upregulate tyrosinase (EC 1.14.18.1), a copper-dependent polyphenol oxidase central to phenolic metabolism. This enzyme catalyzes the ortho-hydroxylation of monophenols and subsequent oxidation to o-quinones, initiating melanogenesis through spontaneous polymerization [2]. Melanin polymers demonstrate broad-spectrum UV attenuation (290–400 nm absorption) while maintaining cellular redox homeostasis, thereby conferring dual photoprotection against direct radiative damage and secondary oxidative injury [3].

*Corallodiscus flabellatus* (Craib.) Burtt., a member of the Gesneriaceae family, is a herbaceous rock plant predominantly found in the alpine scree of Yunnan, Sichuan, and Tibet in China. This species was previously referred to as *C. flabellata* in literature; however, the epithet “*flabellata*” (feminine gender in Latin) does not conform to botanical nomenclature rules and has now been standardized as *C. flabellatus* [4]. In Traditional Chinese Medicine (TCM), the entire plant is esteemed for its properties in clearing damp-heat, detoxifying sores, and delaying the aging process. Prior research has documented the presence of bioactive compounds such as phenylethanoid glycosides (PhGs), flavonoids, and phenolic acids [5]. Notably, the phenylethanoid glycosides have demonstrated a wide range of therapeutic bioactivities, such as anti-inflammatory, antiviral, and neuroprotective properties, thus underscoring their potential as valuable medicinal entities. Specifically, 3,4-dihydroxyphenylethanol-8-O-[4-O-trans-caffeoyl-β-D-apiofuranosyl(1→3)-β-D-glucopyranosyl(1→6)]-β-D-glucopyranoside has been found to have protective effects on the Aβ25–35-induced Alzheimer’s disease (AD) model. The research results indicate that it can improve mitochondrial homeostasis, reduce oxidative stress, and decrease neuronal apoptosis [6].

Given the pivotal role of tyrosinase in mediating UV resistance through phenolic metabolism, this study systematically characterizes the phenolic profile of *C. flabellatus* and elucidates enzyme–substrate interactions. Computational approaches including in silico molecular docking (Autodock Vina 1.2.3) were employed to explicate binding affinities and ligand–enzyme complex stability, providing mechanistic insights into tyrosinase’s catalytic role in alpine plant UV adaptation.

## 2. Results

### 2.1. Structural Elucidation

**Figure 1:**
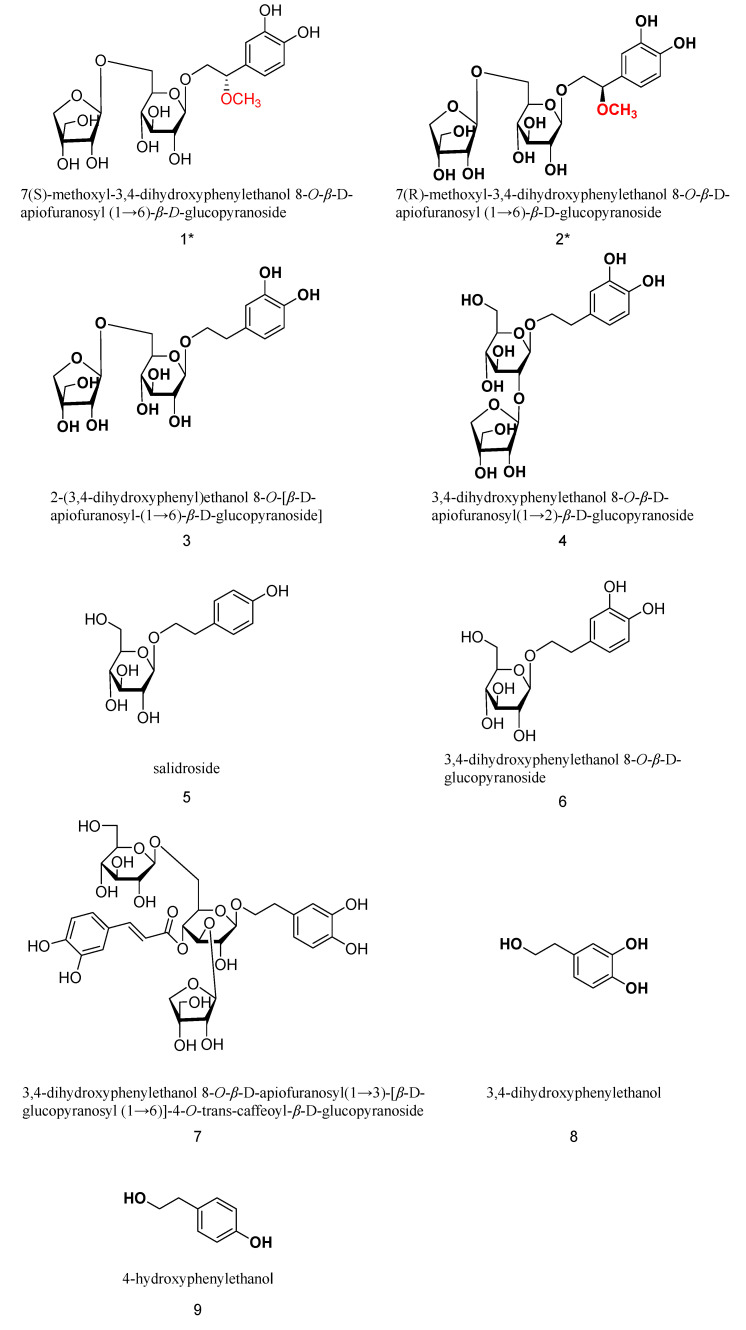
Structures of all compounds (two new compounds marked with *).

The extraction of *C flabellatus* was performed using silica gel, ODS, and preparative HPLC to afford two new phenylethanol glycosides, together with seven known compounds (shown in Figure 1).

Compound **1** is a white amorphous powder that gives a positive reaction with FeCl_3_ reagent, indicating that it is a phenolic compound. Acid hydrolysis on a thin-layer plate detects glucose and apiose, suggesting that the compound is a disaccharide glycoside containing glucose and apiose. The ESI-MS spectrum gives a quasi-molecular ion peak of *m/z*: 477 [M-H]^−^, which, combined with HR-ESI-MS ([M-H]^−^, C_20_H_29_O_13_^−^, found 477.1612, calc. 477.1608), ^13^C-NMR, and DEPT spectra, determines the molecular formula of C_20_H_30_O_13_ and an unsaturation degree of 6. HRESI and NMR spectra of compounds **1** and **2** can be found in Supplementary Materials.

The ^1^H-NMR spectrum shows three hydrogen atoms in the aromatic region, indicating the presence of a phenyl ring. *δ*_H_: 6.69 (d, J = 8.0 Hz, 1H), 6.67 (d, J = 2.0 Hz, 1H), 6.56 (dd, J = 8.0, 2.0 Hz, 1H), suggesting that the phenyl ring is a tri-substituted ABX system. The compound has an unsaturation degree of 6, and besides the phenyl ring, there are two more unsaturated bonds. The signal at *δ*_H_ 3.10 (3H, s) is the hydrogen of the methoxy group. The signals at *δ*_H_: 4.20 (d, J = 7.8 Hz, 1H) and 4.82 (d, J = 3.1 Hz, 1H) indicate the terminal hydrogen of glucose and apiose. Based on this information, it is preliminarily inferred that compound **1** is a disaccharide glycoside containing a phenyl ring, glucose, and apiose.

The ^13^C-NMR spectrum shows a total of 20 carbon signals, including 6 carbons in the phenyl ring, and the remaining 14 carbons should include one glucose (6 carbons), one apiose (5 carbons), one methoxy group, one methyl, and one methylene. The signals at *δ*_C_ 103.4 and 109.2 are the end carbon signals of the two sugars, confirming the existence of two sugars. In addition, the carbon signals of the methylene and methyl groups are at 73.3 and 82.4 ppm, respectively, indicating that they are both oxygenated carbons.

After excluding signals corresponding to the two sugar moieties and the methoxy group, the remaining resonances were attributed to a phenyl ring and two aliphatic carbons, indicating a phenylethanol aglycone skeleton. Compound **3** is a known phenylethanol glycoside isolated from the plant [7]. Comparison of the ^1^H-NMR and ^13^C-NMR of compound **1** with those of compound **3** shows that they are very similar (Table 1). HMBC correlations from H-1′ (δ 4.20) to C-8 (δ 73.5) and from H-1′′ (δ 4.82) to C-6′′ (δ 67.6) displayed characteristic signals for a β-apiofuranosyl-(1→6)-β-glucopyranosyl unit. Additionally, the ^13^C NMR resonance at δ 55.9 (OMe) and HMBC correlations from the methoxy protons (δ 3.10) to C-7 (δ 82.3) established the 7-methoxy substitution on the phenylethanol moiety.

The stereochemistry at the chiral C-7 position was resolved through acid hydrolysis and chiroptical analysis. Compound **1** (10 mg) underwent hydrolysis in 5% H_2_SO_4_ (5 mL) at 90 °C for 12 h. After cooling, the solution was neutralized with 0.1 M Ba(OH)_2_, and the resulting mixture was partitioned between CHCl_3_ and H_2_O. The CHCl_3_ layer afforded aglycone 1a (3.2 mg), identified as (*S*)-2-(3,4-dihydroxy phenyl)-2-methoxyl-ethanol via ^1^H-NMR analysis. The specific rotation of 1a, ${\left[a\right]}_{D}^{20}$ = 41.7° (c 0.02, MeOH), contrasted sharply with similar compounds; (*S*)-(+)-2-(3,4-dihydroxyphenyl)-2-ethoxy-ethanol ([α] = +32.0°) and (*S*)-1-(3,4-dimethoxyphenyl)ethane-1,2-diol ([α] = +36.0°) exhibited comparable values, while (*R*)-1-(3,4-dimethoxyphenyl)ethane-1,2-diol ([α] = –40°) showed an opposite sign [8,9]. This consistency supports the (2*S*)-configuration assignment. Consequently, the absolute configuration of compound **1** was assigned as (7*S*)-7-methoxy-3,4-dihydroxyphenylethanol 8-O-*β*-D-apiofuranosyl-(1→6)-*β*-D-glucopyranoside.

Compound **2** was obtained as a white amorphous powder, showing a phenolic hydroxyl group (FeCl_3_ test positive). Acid hydrolysis followed by TLC analysis (same conditions as in compound **1**) confirmed the presence of D-glucose and D-apiose. HRESIMS displayed a quasi-molecular ion at *m/z* 477.1599 [M–H]^−^ (calcd for C_20_H_29_O_13_^−^, 477.1608), consistent with the molecular formula C_20_H_30_O_13_.

The ^1^H- and ^13^C-NMR data (Table 1) closely resembled those of compound **1**, with key differences in carbon signals [δC 81.7 (C-7), 73.1 (C-8)]. The β-D-apiofuranosyl-(1→6)-β-D-glucopyranosyl unit was confirmed by anomeric protons at δ_H_ 4.15 (d, *J* = 7.8 Hz, H-1′) and δ_H_ 4.85 (d, *J* = 3.0 Hz, H-1″), supported by HMBC correlations from H-1′′ to C-8 (δ_C_ 73.1) and H-1′′ to C-6′′ (δ_C_ 67.6).

Stereochemical resolution via acid hydrolysis yielded aglycone 2a, identified as (*R*)-2-(3,4-dihydroxy phenyl)-2-methoxyl-ethanol by ^1^H-NMR. The optical rotation of 2a, ${\left[a\right]}_{D}^{20}$ = −43.5° (c 0.02, MeOH), confirmed the 7*R* configuration. Thus, compound **2** was characterized as (7*R*)-7-methoxy-3,4-dihydroxyphenylethanol 8-O-*β*-D-apiofuranosyl-(1→6)-*β*-D-glucopyranoside, a stereoisomer of compound **1**.

Furthermore, seven known components from *C. flabellata* were identified to be 2-(3,4-dihydroxyphenyl)ethanol 8-O-[*β*-D-apiofuranosyl-(1→6)-*β*-D-glucopyranoside] (3) [10], 3,4-dihydroxyphenylethanol 8-O-*β*-D-apiofuranosyl(1→2)-*β*-D-glucopyranoside (4) [11], 4-hydroxyphenylethanol 8-O-*β*-D-glucopyranoside (salidroside, 5) [12], 3,4-dihydroxyphenylethanol 8-O-*β*-D-glucopyranoside (6) [13], 3,4-dihydroxyphenylethanol 8-O-*β*-D-apiofuranosyl(1→3)-[ *β*-D-glucopyranosyl (1→6)]-4-O-trans-caffeoyl-*β*-D-glucopyranoside (7) [14], 3,4-dihydroxyphenylethanol (8) [15], and 4-hydroxyphenylethanol (9) [15].

### 2.2. Effects of Phenylethanol Glycosides on Mushroom Tyrosinase

In the tyrosinase inhibition assay (results shown in Table 2), arbutin (positive control) exhibited potent activity with an IC_50_ of 0.15 μM, consistent with literature reports [16]. Compound **7** demonstrated moderate inhibitory effects (IC_50_ = 0.23 μM), while phenylethanol glycosides (1, 2) displayed concentration-dependent biphasic effects—weak inhibition at lower concentrations (10–50 μM) and activation at higher concentrations (100–200 μM). Phenylethanol glycosides (3–6) and their aglycones (8, 9) showed no inhibitory activity but induced significant activation (>120% of baseline activity at 100 μM). The unique inhibitory profile of compound **7** correlates with its structural distinction as the only isolate containing a caffeoyl moiety. This finding is consistent with previous studies showing that the tyrosine analogue of the ortho-dihydroxyphenyl group can act as a substrate for tyrosinase, but the presence of a phenolic unit at the second C6C3 of compound **7** (such as cafoyl) inhibits tyrosinase activity.

### 2.3. Molecular Docking Analysis

Molecular docking studies using AutoDock Vina revealed distinct binding modes of compounds **7**, **1**, and arbutin within the tyrosinase active site. The average binding energy was calculated from three docking runs for 7 (−8.03 ± 0.05 kcal/mol), 1 (−7.17 ± 0.05 kcal/mol), and arbutin (−6.90 ± 0.00 kcal/mol), indicating strong interactions with the enzyme.

**Figure 2:**
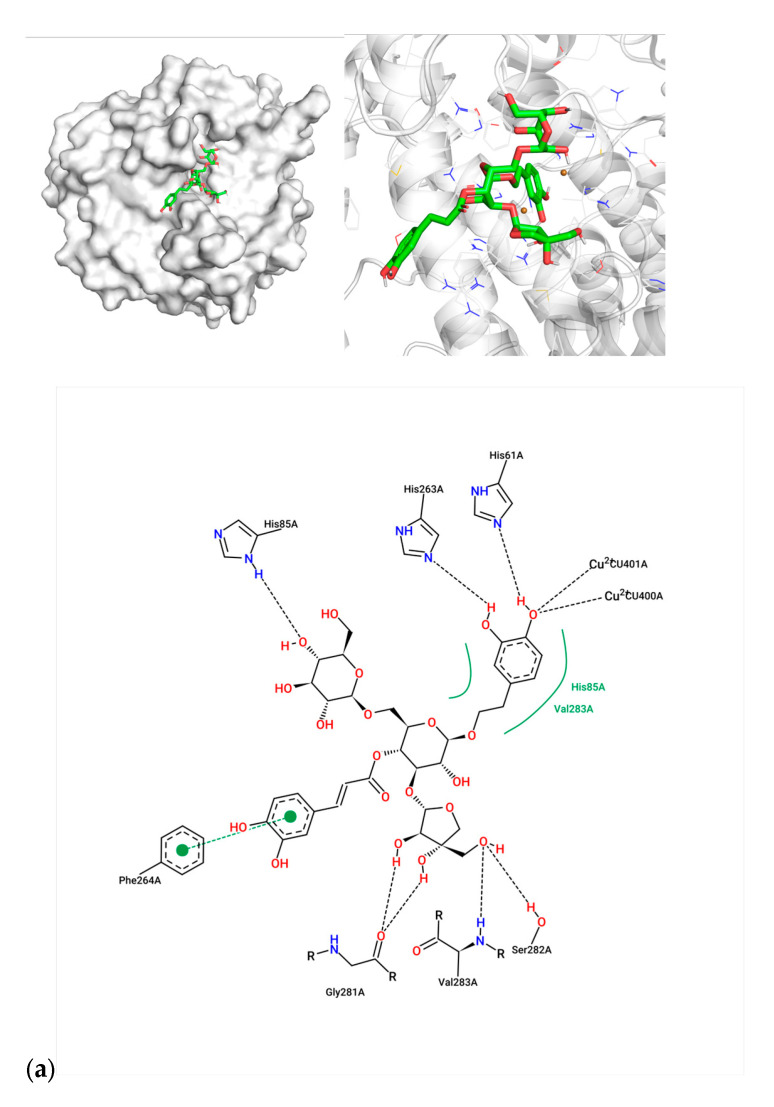

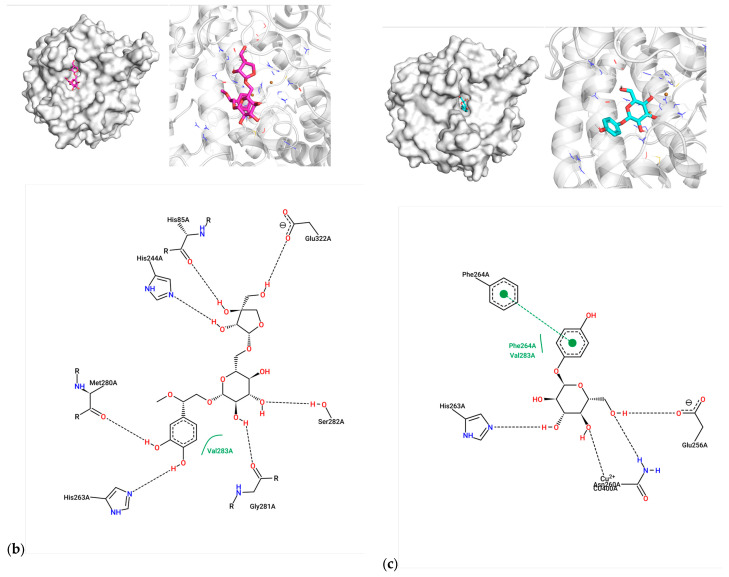
Molecular docking between active compounds: (**a**) compound **7** and 2y9x, (**b**) compound 1 and 2y9x, and (**c**) arbutin and 2y9x.

For compound **7**, the caffeoyl moiety formed π-π stacking interactions with Phe264, while its diphenol structure coordinated with the catalytic Cu^2+^ ions (Figure 2a). Key residues involved included His61, His85, His263, and Val283, consistent with known tyrosinase inhibition mechanisms. Compound **1** exhibited hydrogen bonding with His85, His244, and Ser282, alongside hydrophobic contacts with Met280 and Val283 (Figure 2b). Arbutin, the positive control, bound near Asn260 and Glu256 via hydrogen bonds, with weaker affinity (−6.90 kcal/mol) aligning with its moderate activity (IC_50_ = 0.15 μM) (Figure 2c). The superior inhibitory potency of compound **7** (IC_50_ = 0.23 μM) correlates with its dual interaction mode—metal coordination and aromatic stacking—mirroring features of potent tyrosinase inhibitors.

## 3. Discussion

The phenylethanol glycosides isolated from *C. flabellatus* exhibit structural similarities to melanogenesis substrates. To investigate their inhibitory potential on tyrosinase activity and explore their dermatological applications in melanin suppression and skin whitening, we employed a multidisciplinary approach including tyrosinase activity assays and molecular docking. Intriguingly, the experimental results revealed divergent effects: Compound **7** demonstrated dose-dependent tyrosinase inhibition, whereas compounds **1** and **2** showed biphasic responses (inhibition at low concentrations but activation at high concentrations). Compounds **3**–**6** and **8**–**9** exhibited consistent tyrosinase activation.

Given the structural homology between phenylethanol glycosides and arbutin (a known tyrosinase inhibitor), molecular docking analysis was conducted to elucidate their interaction mechanisms. Contrary to expectations, these glycosides predominantly bound to the active site residues without displaying inhibitory effects, instead potentiating enzymatic activity. Notably, structural analysis of compound **7** revealed that its unique caffeoyl moiety formed stable π-π interactions with Phe264 in the tyrosinase catalytic pocket [17].

## 4. Materials and Methods

### 4.1. Instrument and Materials

One- and two-dimensional NMR spectra were measured using Brucker AV-400 or DRX-500 superconducting nuclear magnetic resonance spectrometers (Bruker Corporation, Karlsruhe, Germany) in deuterated dimethyl sulfoxide (DMSO-d6), deuterated methanol (CD_3_OD), or deuterated acetone (CD_3_COCD_3_). ESI-MS was measured using an API QSTAR Pulsar mass spectrometer (Applied Biosystems Inc., Foster City, CA, USA). Optical rotation was measured using a JASCO DIP-370 digital polarimeter (JASCO Corporation, Tokyo, Japan).

The analytical high-performance liquid chromatography (HPLC) system is the Agilent (Santa Clara, CA, USA) 1200 standard series HPLC with a diode array detector (DAD), manufactured in Germany. The analytical chromatography column is the ZORBAX SB-C18, Agilent, 4.6 mm × 250 mm, 1 mL/min. The semi-preparative chromatography column is the ZORBAX SB-C18, Agilent, 9.4 mm × 250 mm, 3 mL/min. The preparative HPLC is the Agilent 1200 HPLC with a variable-wavelength ultraviolet detector. The preparative chromatography column is the ZORBAX SB-C18, Agilent, 21.2 mm × 150 mm, 15 mL/min. The G and GF_254_ normal-phase silica gel plates are produced by Qingdao Marine Chemical Factory. The normal-phase column chromatography silica gels with 80–100 mesh, 100–200 mesh, and 200–300 mesh, and H (200 mesh) are produced by Qingdao Marine Chemical Factory (Shangdong, China). The MCI-gel CHP-20P (75–150 μm) MCI filling material is produced by Mitsubishi. The hydroxypropyl methylcellulose gel, Sephadex LH-20 (20–100 μm), is produced by Pharmacia. The ODS (YMC*GEL ODS-A, 12 nm, S-50 μm) is produced by YMC.

Tyrosinase mushroom purchased from Sigma has a specific activity of 5037 units/mg. L-tyrosine, DMSO, K_2_HPO_4_, and KH_2_PO_4_ were also purchased from Sigma and are all analytical-grade. Ultrapure water was used for the experiment.

The ELISA reader is a product of MD company in the United States. The pipette is a product of Gilson company in France. The water bath incubator is a product of Shanghai Yiheng Technology Co., Ltd. (Shanghai, China).

### 4.2. Plant Material

In August 2019, the entire plant of *Corallodiscus flabellatus* was collected from Yulong Snow Mountain, Lijiang City, Yunnan Province, with a dry weight of 1 kg. The experimental sample was identified by Professor Xun Gong from the Kunming Institute of Botany, Chinese Academy of Sciences. The voucher specimen (KMUST-B-2019010) is stored in the Key Laboratory of Resource Chemistry of Chinese Materia Medica at Kunming University of Science and Technology.

### 4.3. Extraction and Isolation

A 1 kg sample of dry whole *C. flabellatus* plant was pulverized and soaked in a 70% acetone–water solution at room temperature for 24 h, three times. The extracts were combined and the acetone was removed by vacuum distillation. The residue was then sequentially extracted with petroleum ether, chloroform, and n-butanol, which yielded 9 g of petroleum ether extract, 5 g of chloroform extract, and 50 g of *n*-butanol extract. The n-butanol extract was further purified using Sephadex LH-20 column chromatography, which was eluted with water and gradients of 30%, 60%, 90%, and 100% methanol–water (Fr.1-Fr.6). Using various chromatographic materials and separation techniques such as Sephadex LH-20, MCI gel, ODS, silica gel, and HPLC, nine compounds were isolated and purified.

### 4.4. Spectroscopic Data of the New Compounds

7(*S*)-methoxyl-3,4-dihydroxyphenylethanol 8-*O*-*β*-D-apiofuranosyl (1→6)-*β*-D-glucopyranoside (1): white amorphous powder; ${\left[a\right]}_{D}^{14}$= −26.3° (c = 0.1 g/100 mL, CH_3_OH); ESI-MS: 477 [M-H]^−^; HR-ESI-MS ([M-H]^−^, C_20_H_29_O_13_^−^, found 477.1612, calc. 477.1608). For ^1^H and ^13^C NMR data, see Table 1.

7(*R*)-methoxyl-3,4-dihydroxyphenylethanol 8-*O*-*β*-D-apiofuranosyl (1→6)-*β*-D-glucopyranoside (2): white amorphous powder; ${\left[a\right]}_{D}^{17}$= −57.1° (c = 0.16 g/100 mL, CH_3_OH); ESI-MS: 477 [M-H]^−^; HR-ESI-MS ([M-H]^−^, C_20_H_29_O_13_^−^, found 477.1599, calc. 477.1608). For ^1^H and ^13^C NMR data, see Table 1.

### 4.5. Tyrosinase Inhibition Assay

The inhibitory effects of phenylethanol glycosides on mushroom tyrosinase (EC 1.14.18.1) were evaluated using a 96-well microplate assay adapted from established protocols. Briefly, test compounds (2.0 mg/mL in 10% DMSO-phosphate buffer, pH 6.8) were serially diluted (0–40 μL) and mixed with 40 μL tyrosinase (200 U/mL), 40 μL L-tyrosine (0.1 mM), and 80 μL phosphate buffer (0.1 M, pH 6.8) to a final volume of 200 μL. After incubation at 37 °C for 30 min, dopachrome formation was quantified at 492 nm using a microplate reader. Control wells replaced samples with equivalent DMSO.

### 4.6. Molecular Docking

The crystal structure of *Agaricus bisporus* tyrosinase (PDB ID: 2Y9X) was prepared for docking by removing water molecules and adding polar hydrogens using AutoDock Tools 1.5.7. Ligands, including nine phenylethanol glycosides and arbutin (PubChem CID: 440936), were drawn in ChemDraw 2D v22.0, converted to 3D structures in Chem3D v22.0, and energy-minimized with the MMFF94 force field.

A docking grid of 40 × 40 × 40 Å (1 Å spacing) centered on the active site Cu^2+^ ions (coordinates: x = 12.4, y = 45.8, z = 33.1) was generated to encompass the entire catalytic pocket. AutoDock Vina 1.2.3 was employed with default parameters (exhaustiveness = 8, max poses = 20). To validate the reliability of the molecular docking results, the compound was docked with the protein three times using identical docking parameters, and the average value of the outcomes was calculated. Post-docking analysis of hydrogen bonds and residue interactions was performed using PyMOL and ProteinPlus Server.

## 5. Conclusions

This study elucidates the chemical composition of *Corallodiscus flabellatus* and the tyrosinase-modulating properties of its phenylethanoid glycosides (PhGs), emphasizing their ecological role in high-altitude UV adaptation. Alpine plants face intense UV-B radiation, necessitating defense mechanisms such as melanin synthesis via tyrosinase activity, which mitigates oxidative stress and radiative damage. Our investigation revealed that PhGs exhibit divergent regulatory effects on tyrosinase, reflecting adaptive strategies under environmental extremes.

Two new PhGs and seven known compounds were identified. Biological assays demonstrated distinct activity patterns: compounds **3**–**6** and their aglycones consistently enhanced tyrosinase activity, whereas compound **7** displayed potent inhibition, comparable to established inhibitors. Notably, two new PhGs exhibited concentration-dependent dual effects—weak inhibition at lower concentrations and marked activation at higher levels. Structural analysis revealed that the inhibitory activity of compound **7** stemmed from its caffeoyl moiety, which stabilized interactions with the enzyme’s active site through aromatic stacking and metal coordination. In contrast, activating PhGs likely function as substrates, promoting enzymatic turnover.

The observed tyrosinase activation aligns with alpine plants’ need to upregulate melanogenesis for UV protection, as melanin absorbs harmful radiation and neutralizes reactive oxygen species. Conversely, the inhibitory effect of compound **7** suggests a regulatory mechanism to prevent oxidative overload during prolonged UV exposure. This dual modulation, involving activation for acute stress response and inhibition of homeostasis, highlights an evolutionary balance critical for survival in extreme environments.

## Figures and Tables

**Table 1:** Hydrogen–carbon spectrum data for compounds **1** and **2** (500 MHz, DMSO-d6) δ in ppm.

No.	Compound 1		Compound 2	
	^13^C	^1^H	^13^C	^1^H
1	129.9		129.8	
2	115.4	6.67 (1H, d, J = 2.0 Hz)	115.4	6.69 (1H, overlap)
3	145.2		145.2	
4	144.9		145.0	
5	114.0	6.69 (1H, d, J = 8.0 Hz)	114.2	6.68 (1H, d, J = 8.1 Hz)
6	118.0	6.56 (1H, dd, J = 8.0, 2.0 Hz)	118.1	6.57 (1H, dd, J = 8.1, 2.3 Hz)
α(8)	73.5	3.57, 2.93 (each 1H, overlap)	73.1	3.58, 2.94 (each 1H, m)
β(7)	82.3	4.22 (1H, m)	81.7	4.21 (1H, dd, J = 8.1, 3.5 Hz)
1′	103.3	4.20 (1H, d, J = 7.8 Hz)	102.8	4.15 (1H, d, J = 7.8 Hz)
2′	73.2	3.57 (1H, overlap)	73.2	3.78 (1H, m)
3′	75.4	3.24 (1H, t, J = 6.9)	75.5	3.23 (1H, t, J = 8.3 Hz)
4′	70.1	2.96 (1H, m)	70.1	2.96 (1H, m)
5′	76.6	3.12 (1H, overlap)	76.6	3.12 (1H, m)
6′	67.6	3.84, 3.34 (each 1H, overlap)	67.7	3.85, 3.32 (each 1H, m)
1″	109.1	4.82 (1H, d, J = 3.1 Hz)	109.2	4.85 (1H, d, J = 3.0 Hz)
2″	75.8	3.74 (1H, d, J = 3.1 Hz)	75.8	3.75 (1H, d, J = 3.2 Hz)
3″	78.8		78.8	
4″	73.2	3.83, 3.57 (each 1H, overlap)	73.1	3.83, 3.56 (each 1H, m)
5″	63.1	3.30 (2H, overlap)	63.1	3.33 (2H, s)
7-OCH_3_′	55.9	3.10 (3H, s)	55.8	3.09 (3H, s)

**Table 2:** Effects of benzyl alcohol and glycoside compounds on mushroom tyrosinase.

		Compound	Arbutin	1	2	3	4	5	6	7	8	9
	Inhibition Rate											
Concentration (mg/mL)												
0.05			0.46	0.27	0.11	−0.29	−0.02	−0.98	−0.52	0.11	−2.05	−1.40
0.1			0.62	0.11	0.11	−0.64	−0.10	−1.08	−0.64	0.29	−2.42	−0.84
0.2			0.90	0.11	0.07	−0.78	−0.69	−0.96	−0.80	0.46	−1.42	−0.78
0.3			0.86	−0.04	0.02	−0.67	−1.00	−0.71	−0.54	0.76	−0.98	−0.80
0.4			0.97	−0.22	−0.24	−0.60	−0.60	−1.29	−0.60	0.71	−0.90	−0.57

The concentration of the sample was mg/mL.

## Data Availability

The original contributions presented in this study are included in the article. Further inquiries can be directed to the corresponding author.

## Supplementary Materials

The following supporting information can be downloaded at https://www.mdpi.com/article/10.3390/molecules30112296/s1: Figures S1–S17: key HRESI and NMR spectra of compounds 1 and 2. Figure S18: Calculation of the IC_50_ of Compound **7**.
