# Supplementary material for: Chemical Profiling and Tyrosinase Inhibition Mechanism of Phenylethanoid Glycosides from Corallodiscus flabellatus
Source: Molecules. 2025 May 23;30(11):2296. doi: 10.3390/molecules30112296 (PMC12156022; doi:10.3390/molecules30112296)
Supplement: Supplementary file 1 [file molecules-30-02296-s001.zip › molecules-3576332-supplementary.pdf]

*Supplementary materials*

# Chemical Profiling and Tyrosinase Inhibition Mechanism of Phenylethanoid Glycosides from *Corallodiscus flabellatus*

**Hong-bo Deng, Yao Yao and Hai-zhou Li ***

Faculty of Life Science and Technology, Kunming University of Science and Technology, 727 Jingming South Road, Chenggong District, Kunming 650500, China
* Correspondence: Correspondence: lihaizhou@kust.edu.cn; Tel.: +86-871-65920253

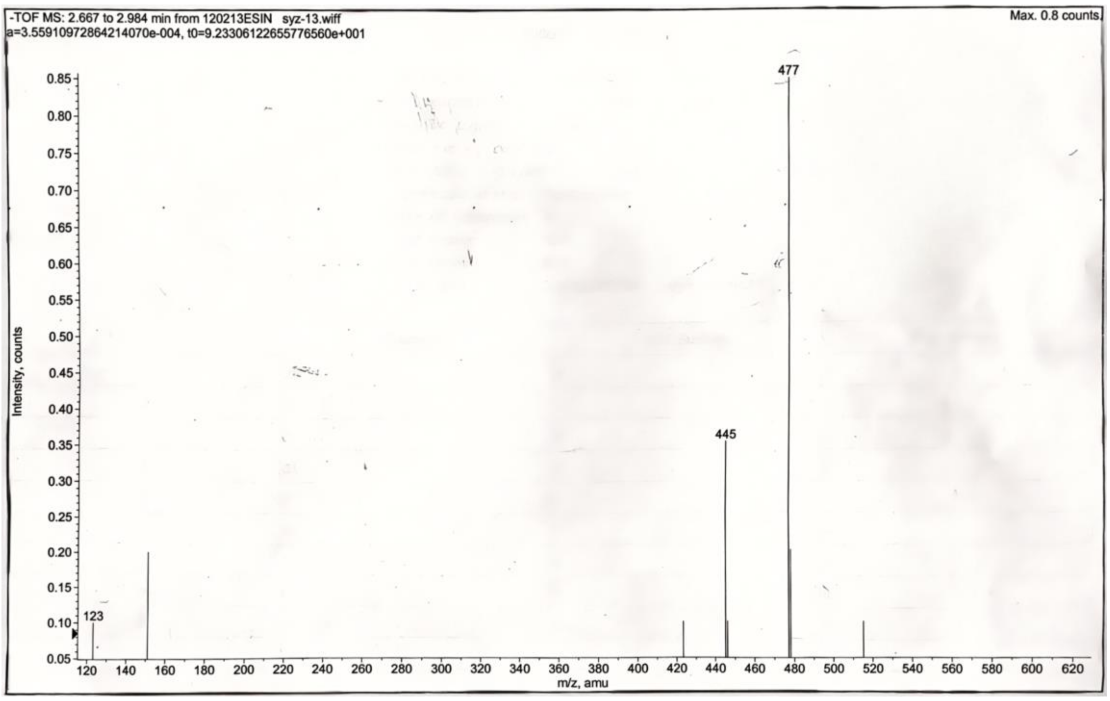


**Figure S1.** ESI spectrum of compound **1.**

Elemental composition calculator

```
Target m/z:      +477.1612      amu
Tolerance:         +10.0000     ppm
Result type:   Elemental
Max num of results:    1000
Min DBE:         -10.0000      Max DBE:          +60.0000
Electron state:        OddAndEven
Num of charges:        0
Add water:     N/A
Add proton:    N/A
```

**Figure S2.** HRESIMS spectrum of compound **1.**

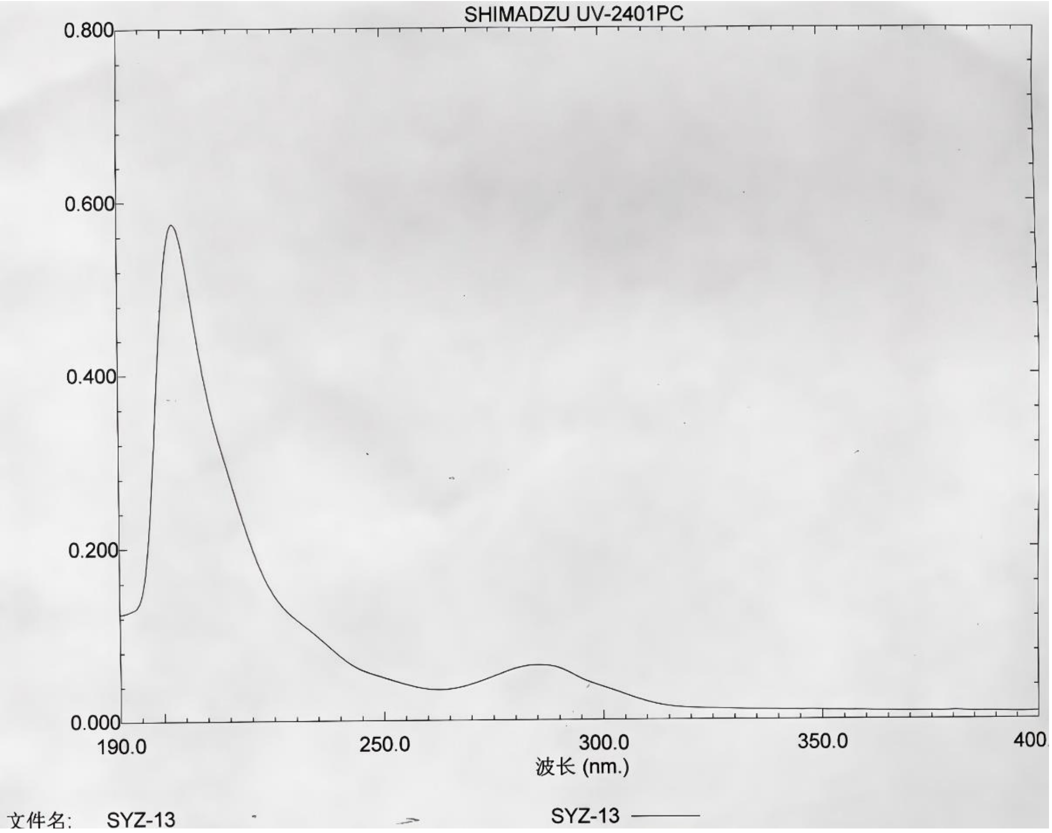


**Figure S3.** UV spectrum compound **1.**

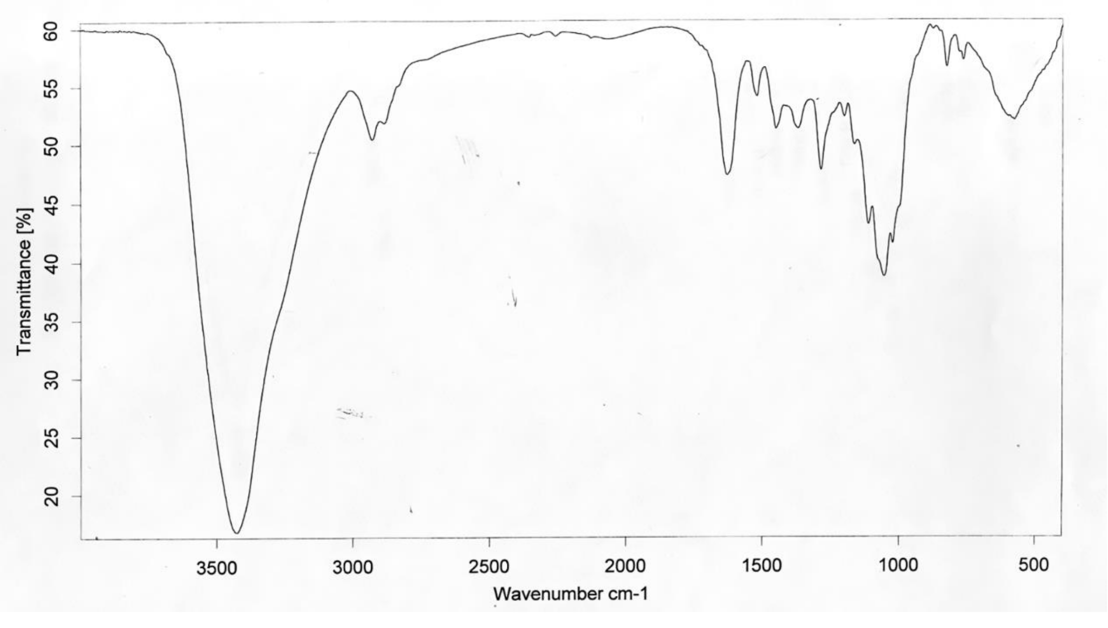


**Figure S4.** IR spectrum of compound **1.**

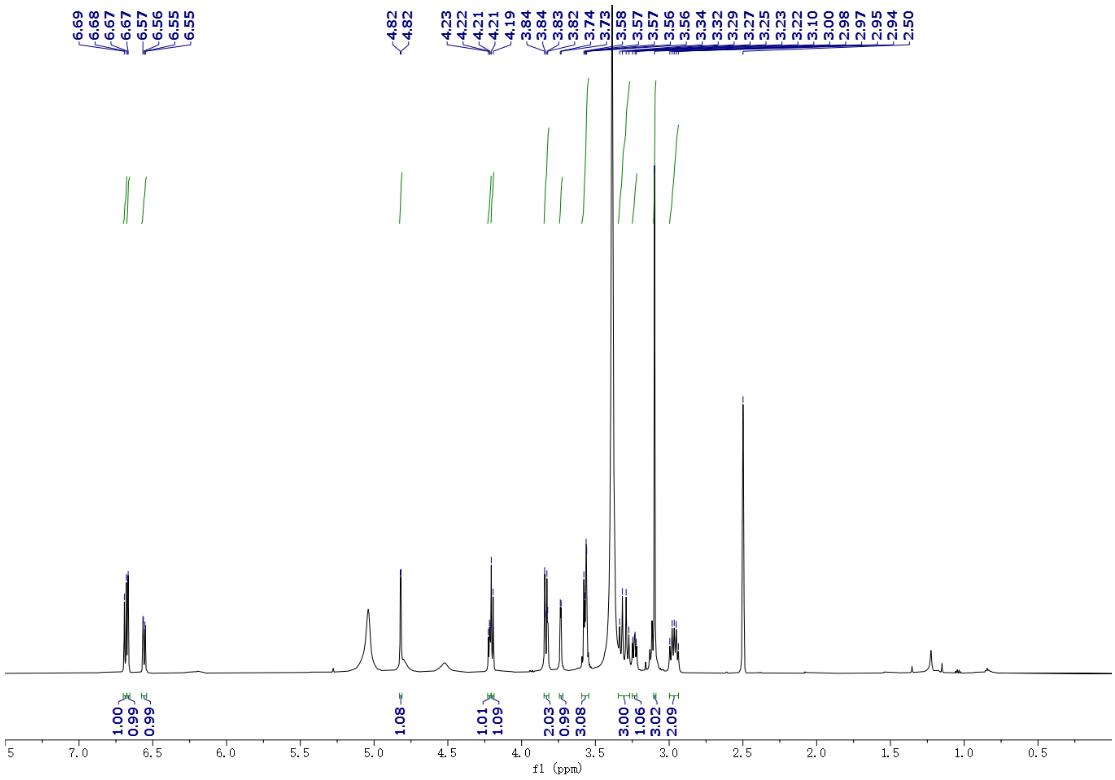


**Figure S5.** $^{1}H$ NMR spectrum (600 MHz) of compound **1** in DMSO.

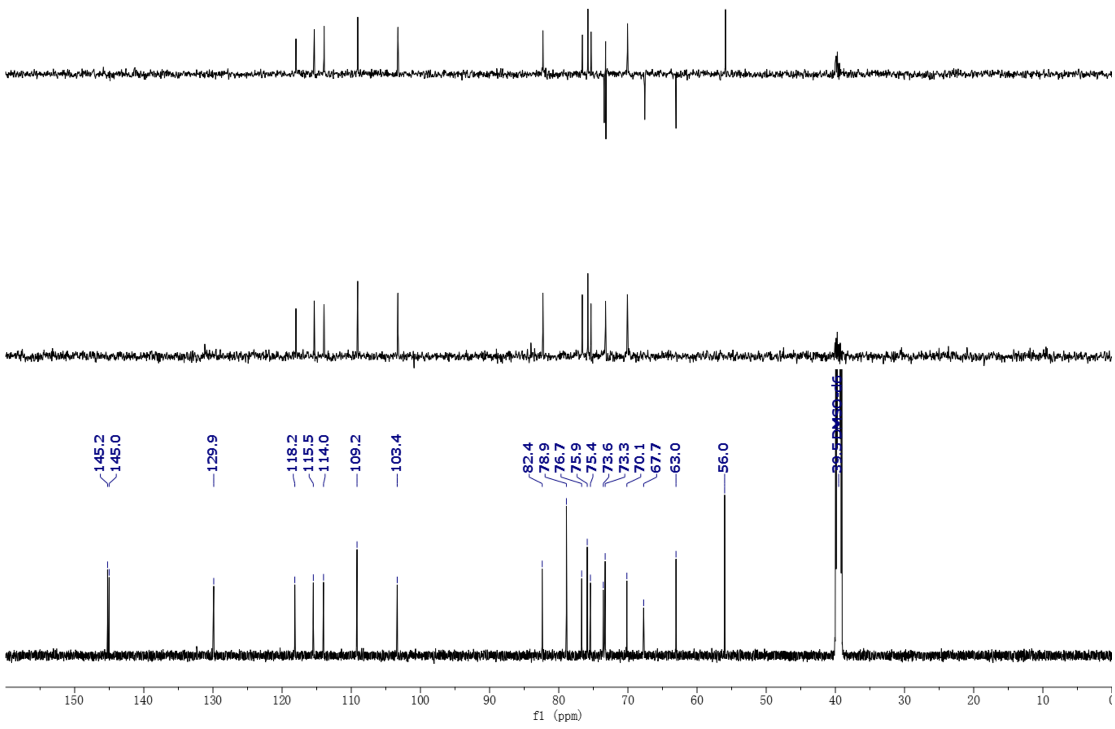


**Figure S6.** $^{13}C$ NMR spectrum (150 MHz) of compound **1** in DMSO.

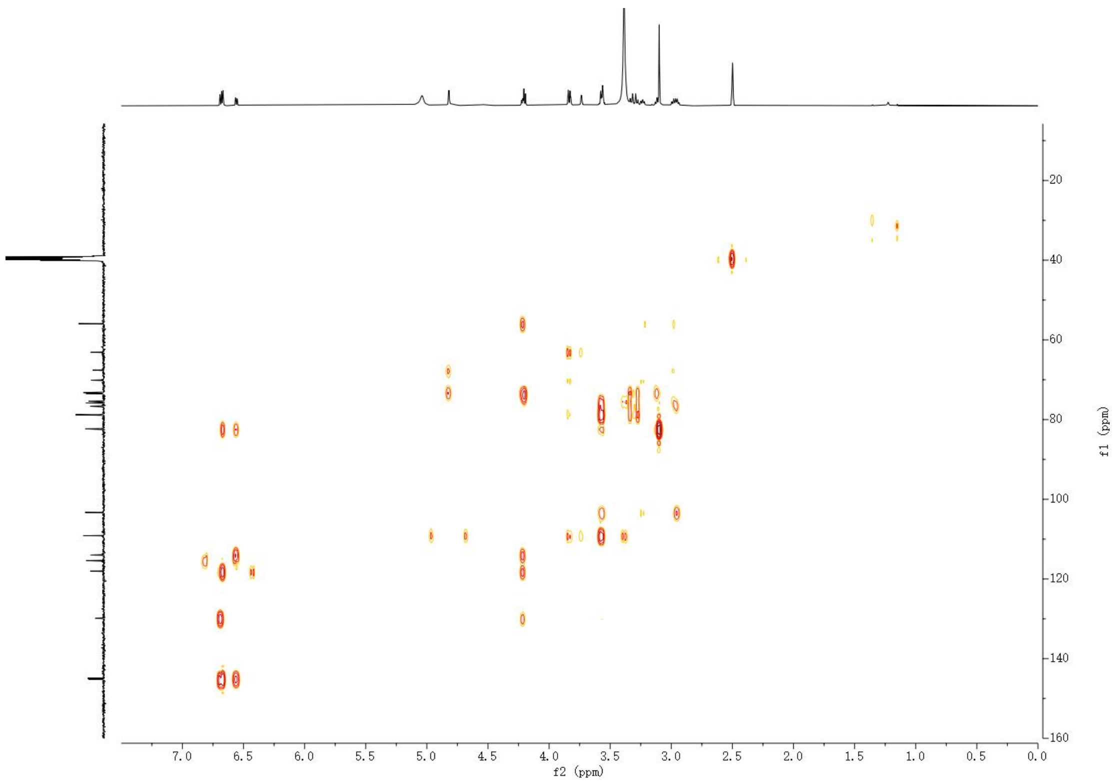


**Figure S7.** HMBC spectrum of compound **1** in DMSO.

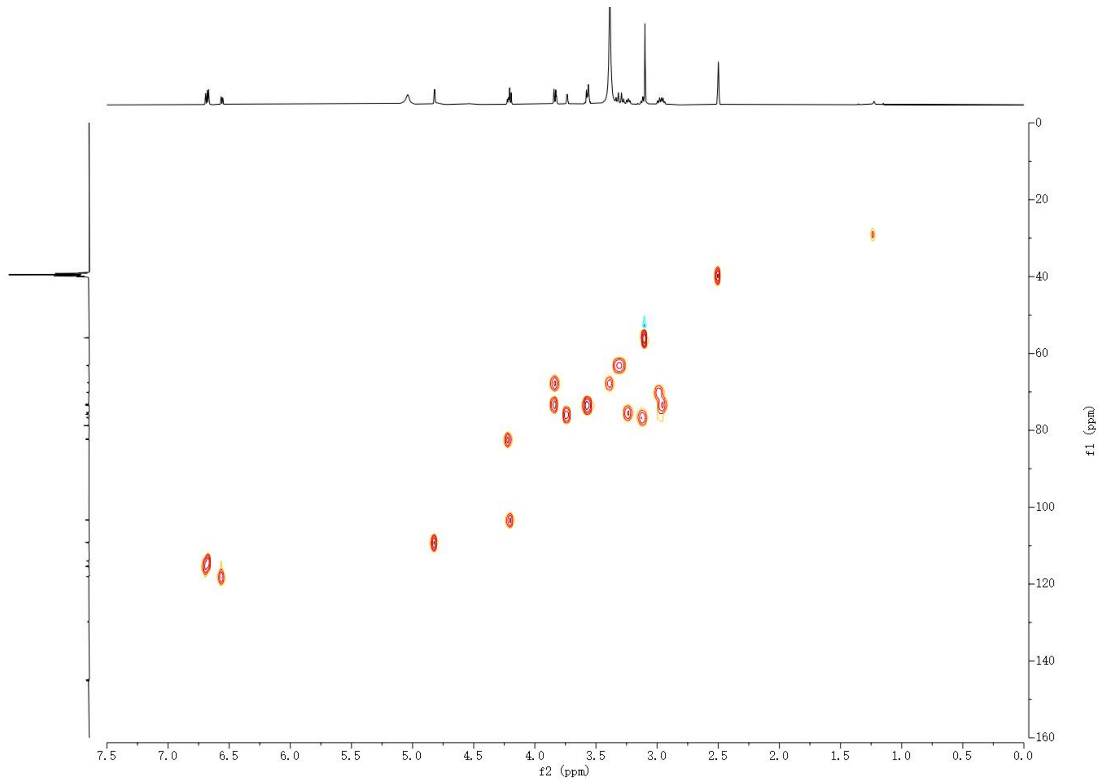


**Figure S8.** HSQC spectrum of compound **1** in DMSO.

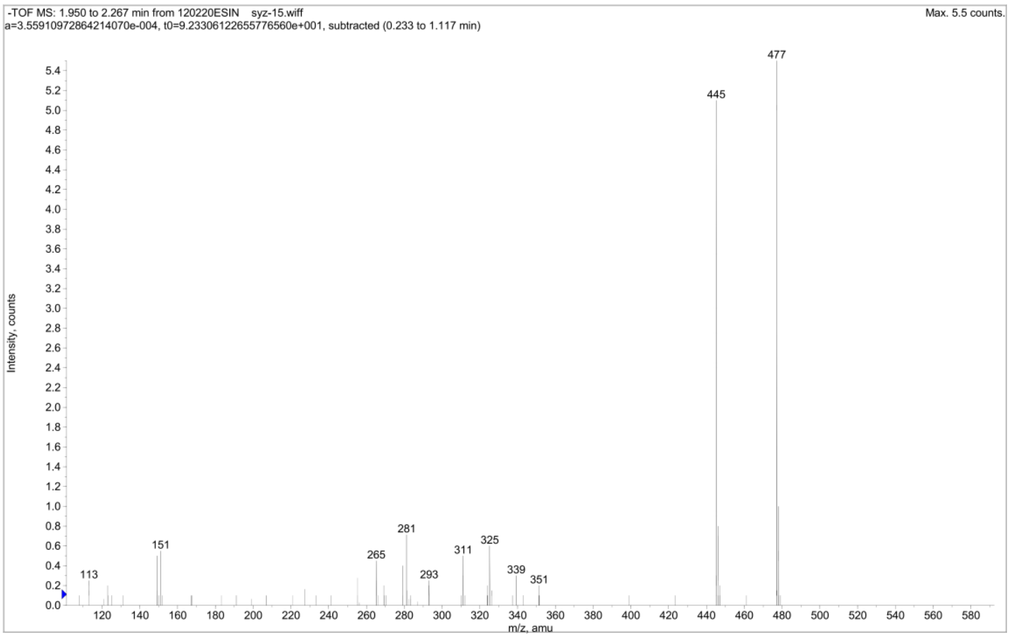


**Figure S9.** ESI spectrum of compound **2.**

# Elemental composition calculator

```
Target m/z:    +477.1599      amu
Tolerance:      +10.0000      ppm
Result type:   Elemental
Max num of results:   1000
Min DBE:        -10.0000      Max DBE:          +60.0000
Electron state:        OddAndEven
Num of charges:        0
Add water:     N/A
Add proton:    N/A
```

**Figure S10.** HRESIMS spectrum of compound **2.**

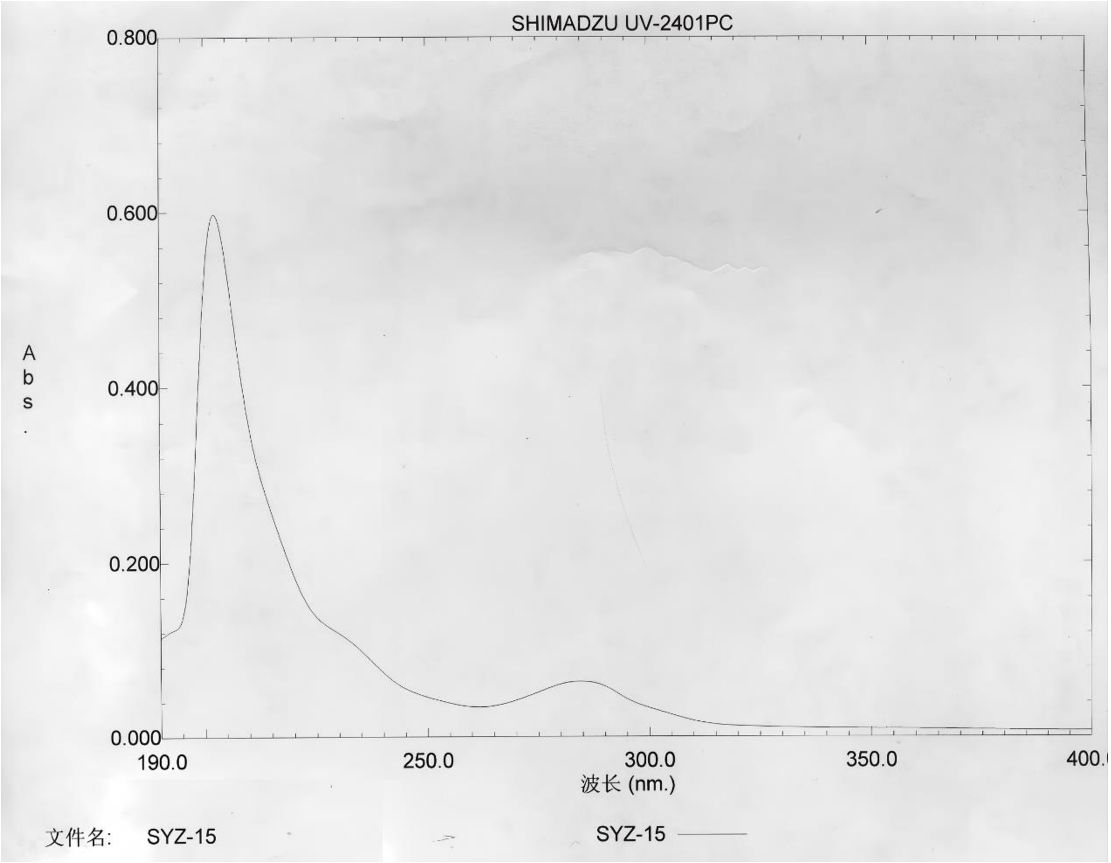


**Figure S11.** UV spectrum of compound **2.**

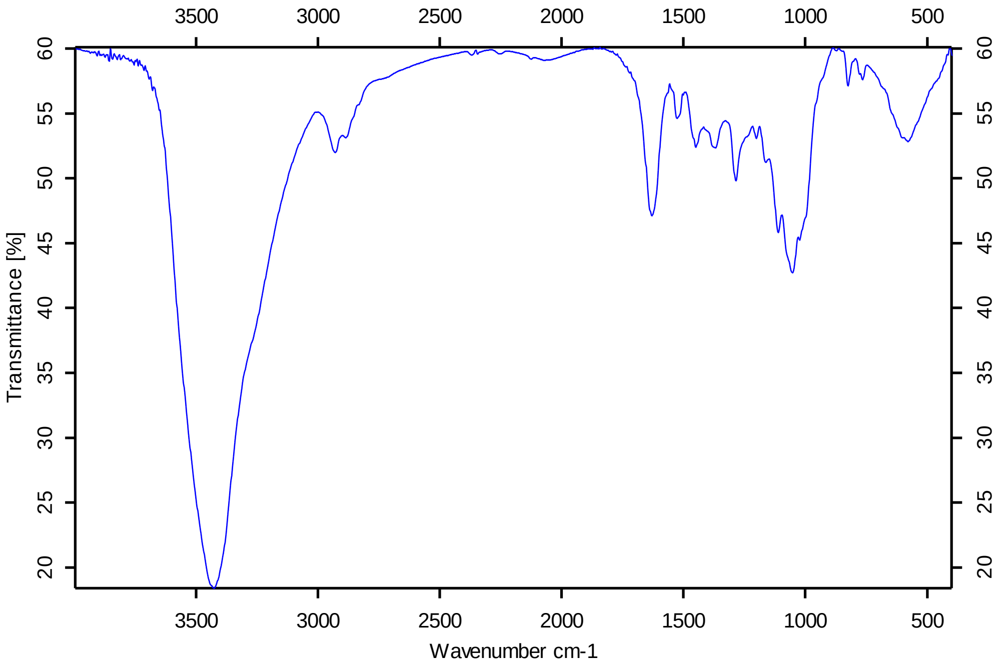


**Figure S12.** IR spectrum of compound **2.**

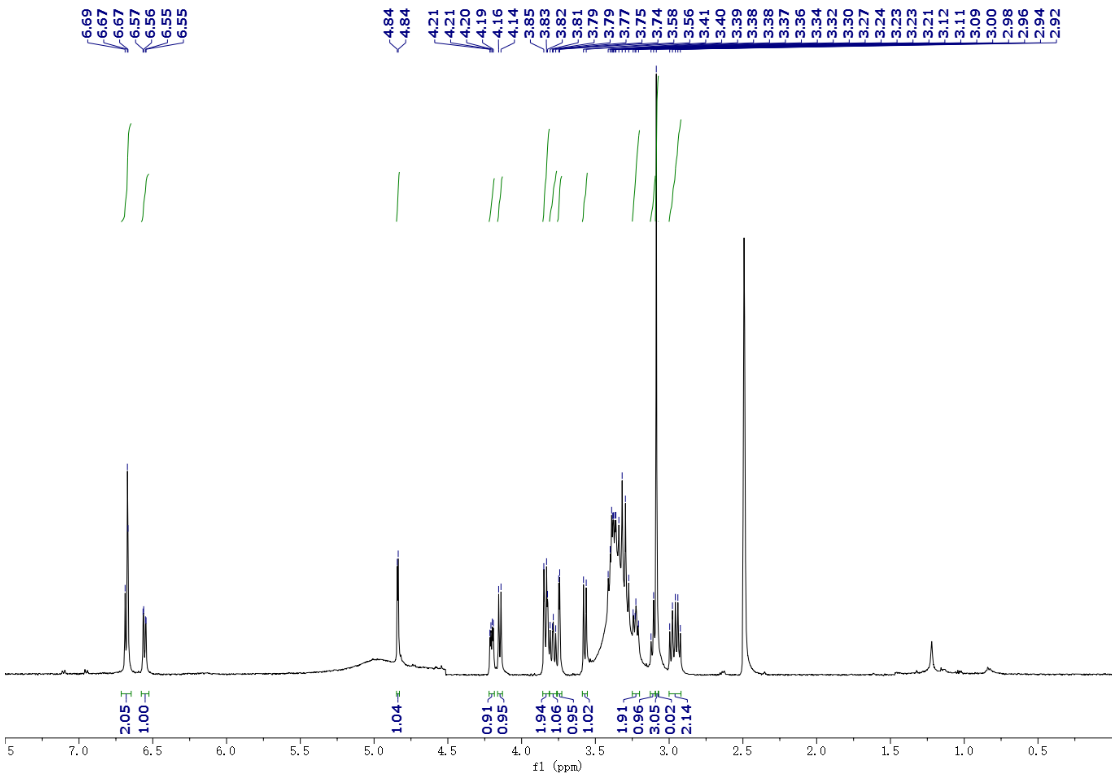


**Figure S13.** $^{1}$H NMR spectrum (600 MHz) of compound **2** in DMSO.

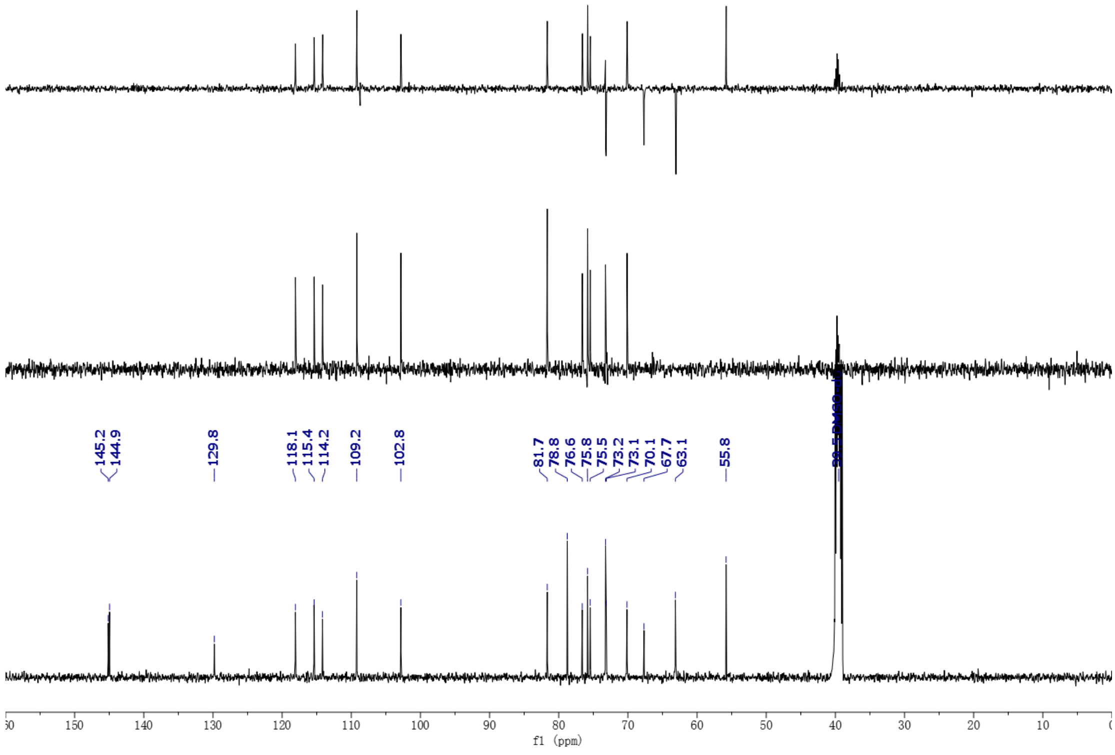


**Figure S14.** $^{13}$C NMR spectrum ( 150 MHz) of compound **2** in DMSO.

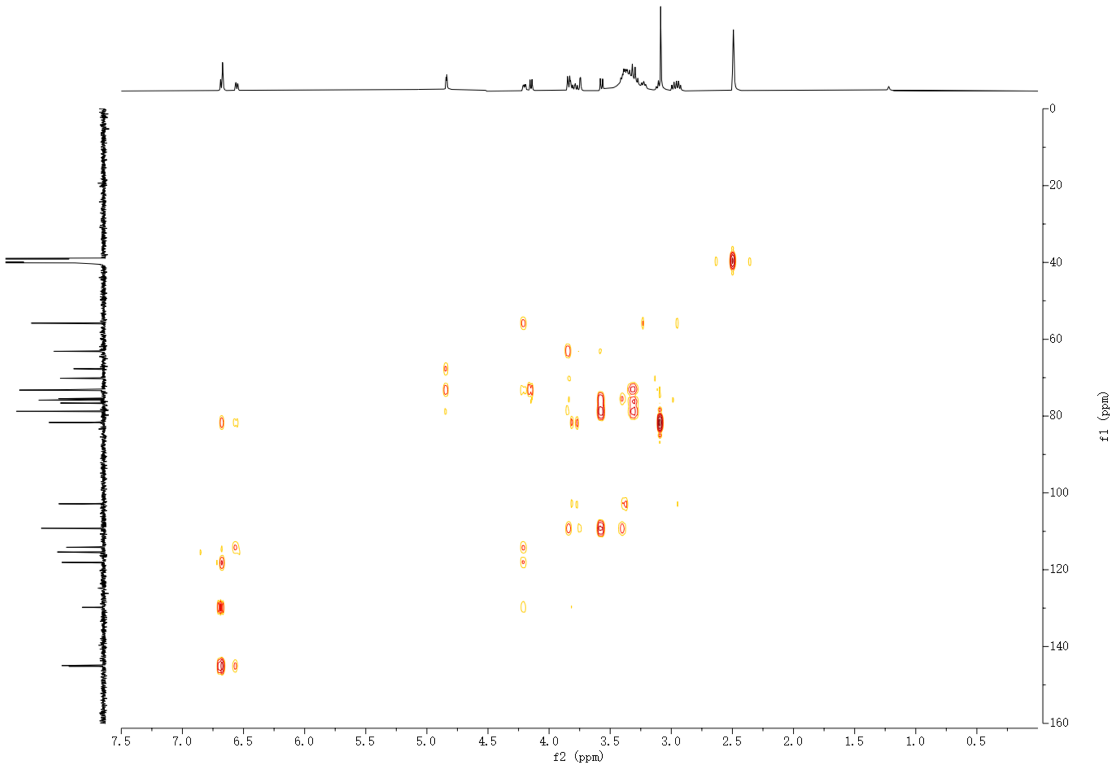


**Figure S15.** HMBC spectrum of compound **2** in DMSO.

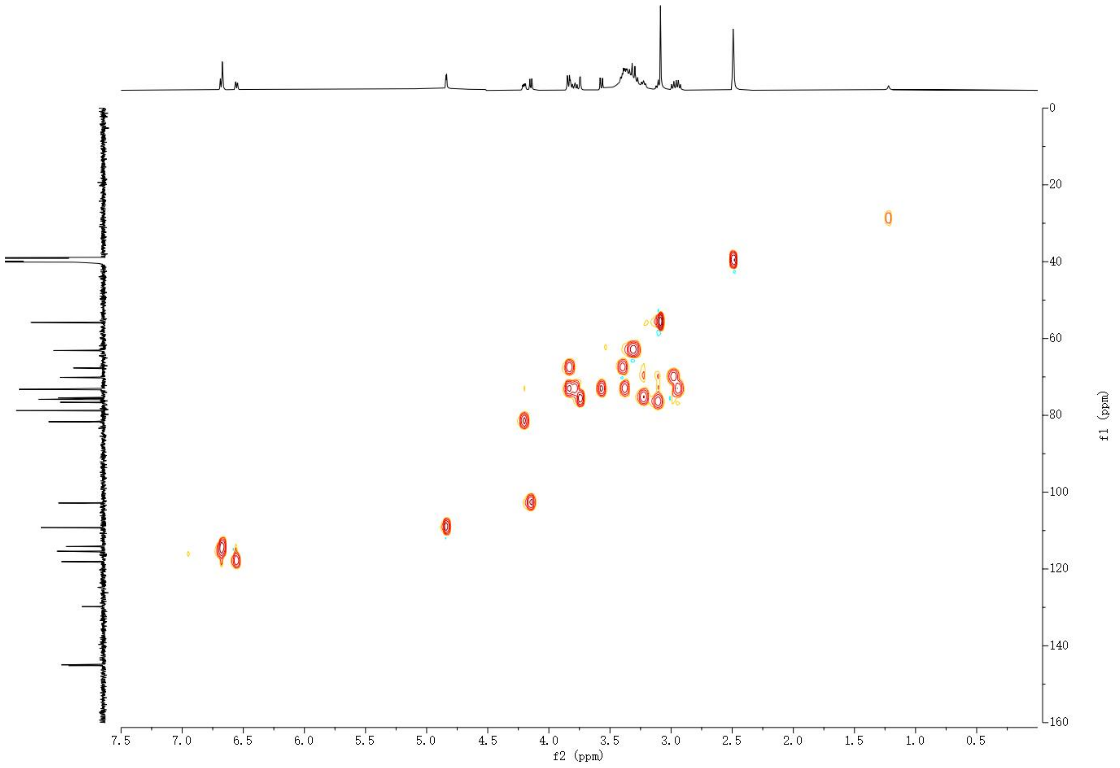


**Figure S16.** HSQC spectrum of compound **2** in DMSO.

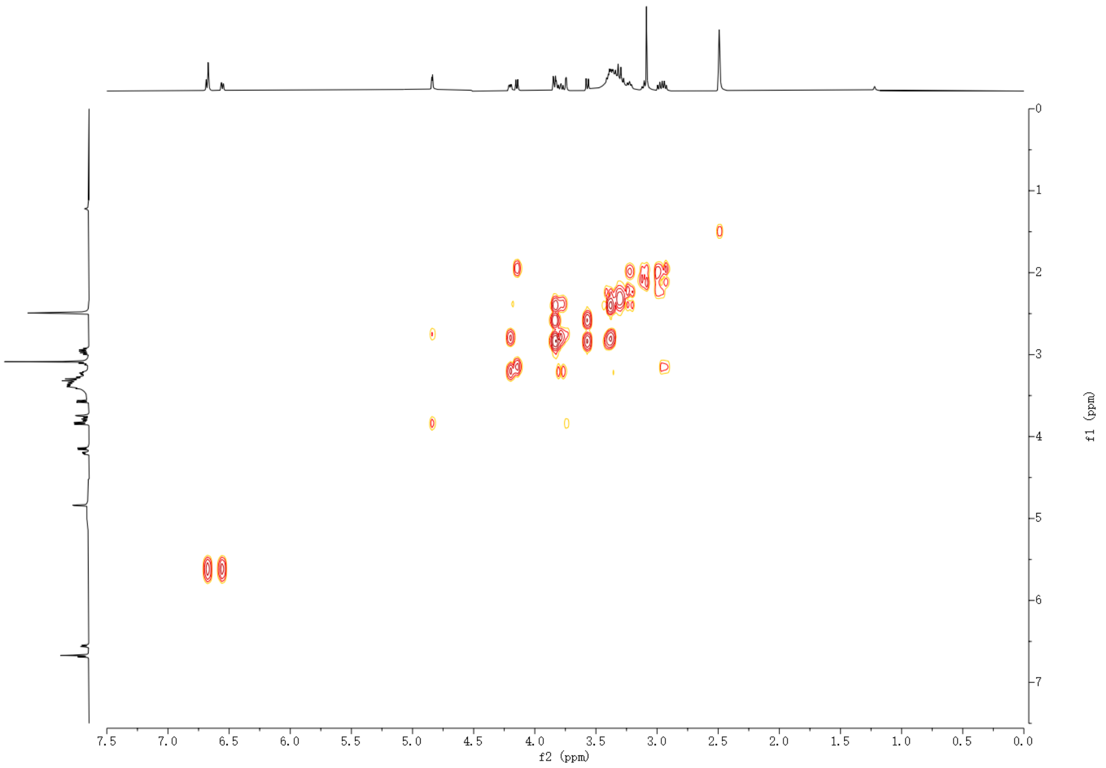


**Figure S17.** $^1$H-$^1$H COSY spectrum of compound **2** in DMSO.

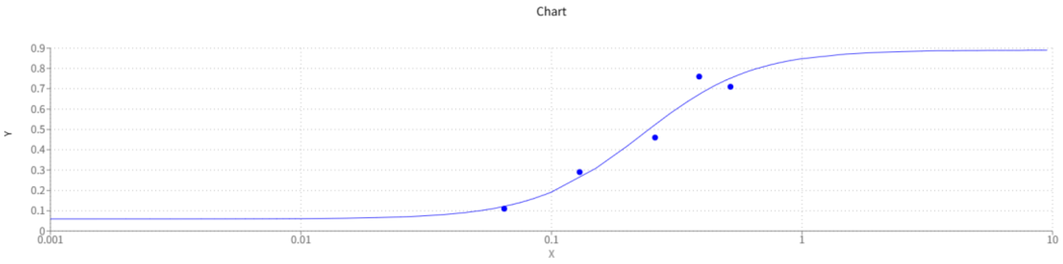


$$Y = 0.0597 + \frac{0.8903 - 0.0597}{1 + \left(\frac{X}{0.2304}\right)^{-1.9896}}$$

IC50=0.23uM

**Figure S18.** Calculation of the $IC_{50}$ of Compound **7.**
